# Reduced Graphene Oxide Modulates the FAK-Dependent Signaling Pathway in Glioblastoma Multiforme Cells In Vitro

**DOI:** 10.3390/ma15175843

**Published:** 2022-08-24

**Authors:** Jaroslaw Szczepaniak, Malwina Sosnowska, Mateusz Wierzbicki, Olga Witkowska-Pilaszewicz, Barbara Strojny-Cieslak, Joanna Jagiello, Wiktoria Fraczek, Marcin Kusmierz, Marta Grodzik

**Affiliations:** 1 Department of Nanobiotechnology, Institute of Biology, Warsaw University of Life Sciences (WULS-SGGW), 02-787 Warsaw, Poland; 2 Department of Large Animal Diseases and Clinic, Institute of Veterinary Medicine, Warsaw University of Life Sciences, 02-787 Warsaw, Poland; 3 Graphene and Composites Research Group, Łukasiewicz Research Network-Institute of Microelectronics and Photonics, 01-919 Warsaw, Poland; 4 Analytical Laboratory, Institute of Chemical Sciences, Faculty of Chemistry, Maria Curie-Skłodowska University, 3 Maria Curie-Skłodowska Square, 20-031 Lublin, Poland

**Keywords:** graphene, reduced graphene oxide, glioblastoma, FAK, β-catenin, cell mobility, invasiveness, migration, cell membrane receptors, U87 cell line

## Abstract

Aggressive invasiveness is a common feature of malignant gliomas, despite their high level of tumor heterogeneity and possible diverse cell origins. Therefore, it is important to explore new therapeutic methods. In this study, we evaluated and compared the effects of graphene (GN) and reduced graphene oxides (rGOs) on a highly invasive and neoplastic cell line, U87. The surface functional groups of the GN and rGO flakes were characterized by X-ray photoelectron spectroscopy. The antitumor activity of these flakes was obtained by using the neutral red assay and their anti-migratory activity was determined using the wound healing assay. Further, we investigated the mRNA and protein expression levels of important cell adhesion molecules involved in migration and invasiveness. The rGO flakes, particularly rGO/ATS and rGO/TUD, were found highly toxic. The migration potential of both U87 and Hs5 cells decreased, especially after rGO/TUD treatment. A post-treatment decrease in mobility and FAK expression was observed in U87 cells treated with rGO/ATS and rGO/TUD flakes. The rGO/TUD treatment also reduced β-catenin expression in U87 cells. Our results suggest that rGO flakes reduce the migration and invasiveness of U87 tumor cells and can, thus, be used as potential antitumor agents.

## 1. Introduction

Gliomas are the most common primary malignancy of the central nervous system [1]. Stage IV glioblastoma (GBM) is one of the most aggressive and lethal types of cancer, with an average survival time of 15 months from diagnosis [2], GBM is characterized by diffusive growth hindering complete resection, genetic changes conditioning immunity to apoptosis, high proliferation and invasion, and poor responses to current therapy. Standard treatment is limited and consists of surgical resection of the tumor followed by chemotherapy and radiotherapy. The most commonly used chemotherapy is the oral alkylating agent, temozolomide [3]. All these features contribute to relapse of the neoplastic disease and a very poor prognosis for the patient [4].

Moreover, the common feature of gliomas is high invasiveness, despite strong biological heterogeneity [5]. Thus the development of the new effective therapies is a very important issue. The distinction between migration and invasiveness is essential. Migration is mostly limited to certain stages of cell cycle. In addition, it is a physiological reaction performed by many cell types, for example by neural stem cells (NSCs), which budge along the brain tissue boundary. This process is regulated by highly invasive cells. In addition, tissue damage can occur in both cases, and invasion is an undesirable, anatomically inadequate, and a non-physiological reaction [6]. Several complex cellular and molecular processes which are connected with the damage of extracellular matrix (ECM) components, cell separation, and diapedesis through the basement membrane and stroma influence tumor cell invasion. ECM degradation is due to overexpression of key proteases co-related to increased invasion (e.g., uPA, uPAR, cathepsin B, MMP, and ADAM), whose activity in healthy tissue is controlled by endogenous inhibitors (e.g., PAI1, PAI2, cystatin C, stefin A, stefin B), which may not function properly in neoplastic cells [7,8].

Moreover, fibronectin, the dominant component of the extracellular matrix (ECM), is involved in the development of GBM intrusion. GBM shows increased invasion activity in the presence of fibronectin [9]. However, recently it was confirmed that the upregulation of fibronectin (FNMA) may result in reduced mobility and tumor cell aggressiveness [10]. They are likely made possible by increased cell–cell and cell–ECM adhesion abilities. This process may be connected with the upregulation of α5 integrin expression, which can be activated by dexamethasone [6]. ECM transition is a highly regulated process that requires a balance between cell connections as well as between annihilation and remodeling of the ECM. Interactions between ECM and individual cells are essential in attributing the nature of invasiveness. Many different brain ECM components may influence the creation of a favorable migration environment for GBM cells [11]. Many receptors are involved in the process of invasiveness, including integrins, which are involved in cell–cell and cell–matrix interactions. For example, in GBM, the β1 integrin is usually highly expressed, which activates an invasiveness of the GMB cells [12]. The α5 subunit of the integrins may bind to fibronectin, one of the extracellular ligands, which leads to an activation of intracellular signaling cascades influencing an increased cell motion [9]. In addition, cadherins are believed to be important cellular components because these transmembrane proteins influence the creation of the adjacent junctions (AJs) in cell–cell adhesion by, for example, indirectly regulating apoptosis, gene expression, and cell proliferation and migration [13,14,15,16]. In addition, cadherins also take part in cell–cell attachment by N-cadherin downregulation, which influences cell polarity and motion, with significant increases in tumor cell migration and invasiveness [17]. Other researchers have demonstrated that aggressiveness of GMB correlates with N-cadherin expression and is connected with the Ki-67 labeling index, thus influencing cell proliferation and differentiation [18]. Moreover, some protein tyrosine kinases play a key role in migration, proliferation, and survival of several different cell types [19]. One of them, called FAK (focal adhesion kinase), is activated by ligand binding and clustering of cell-surface integrin receptors [20]. It promotes neoplasm progression and metastasis by affecting the tumor microenvironment. Some FAK functions include control of cell motion, invasiveness, gene expression, and self-renewal of tumor stem cells [21]. Because all aforementioned molecules are related to migration and invasiveness mechanisms, and the invasion of GBM cells is one of the biggest challenges associated with the therapeutic management of GBM, it seems justified to study the influence of new materials, such as graphene (GN) and reduced graphene oxides (rGO) flakes in our case. 

Therefore, when it comes to rGO flakes, a crucial discovery may be the reduction in the content of oxygen-containing functional groups that may disrupt signaling pathways in the cell membrane or directly interact with cellular structures such as integrin α5, integrin β1, β-catenin, N-cadherin, PAN-cadherin, and FAK in comparison to GO. Fiorillo et al. demonstrated that the presence of multiple oxygen-containing functional groups in GO favors an adhesion to U87 tumor cells [22]. We previously showed that the allotropic forms of carbon (C60, nfND) could cause cell cycle arrest and thus reduce cell proliferation and virulence as well as the invasion of the hepatocellular carcinoma cell lines HepG2 and C3A [23,24]. We also showed that the use of the nfGO and CELE cocktail (chicken embryo liver extract) reduced proliferation by arresting the cell cycle, increasing the expression of adhesion genes such as FAK, E-cadherin, and N-cadherin, and decreasing β-catenin expression [24]. Further, we showed that ND (nano diamond), NG (nano graphene), and nGO (nano graphene oxide) nanoparticles reduced the adhesion and invasiveness of U87 and U118 GBM cell lines and thereby affected the activity of the EGFR/AKT/mTOR and β-catenin pathways [25]. These results indicate that the tested carbon allotropes may act as potential anticancer therapeutic agents by inhibiting cell proliferation and invasion. Therefore, in this study, we analyzed the influence of other allotropic forms of carbon, including rGO, on the phenomenon of adhesion and invasiveness in neoplastic cells of glioblastoma multiforme. The main aim of the study was to determine the effect of rGO/ATS and rGO/TUD flakes in comparison with GN/ExF in exerting modulating and transducing effects on the mechanisms of adhesion and related invasiveness in U87 glioblastoma multiforme cells. For this purpose, migration and mobility assessment tests were used, as well as molecular analyses examining the expression of adhesion and invasiveness markers [α5 integrin, β1 integrin, β-catenin, N-cadherin, PAN-cadherin, and FAK (focal adhesion kinase)] responsible for cell–cell and cell–ECM junctions, both at the mRNA and protein levels. 

## 2. Materials and Methods

### 2.1. GN and rGO Specimens

The Łukasiewicz Research Network—Institute of Microelectronics and Photonics, Warsaw prepared the GN and rGO. To obtain the GN/ExF graphene flakes, direct graphite exfoliation using Capstone (a fluorinated surfactant) was adopted. The graphite oxidation and exfoliation were used for obtaining the GO, according to a modified Marcano method. Marcano et.al. found that excluding NaNO3 increases the amount of KMnO4 and performs the reaction in a 9:1 mixture of H2SO4/H3PO4, which improves the efficiency of the oxidation process. This improved method provides a greater amount of hydrophilic oxidized graphene material. Moreover, the GO produced by Marcano et al.’s method is more oxidized than that prepared by Humme et al.’s method and does not generate toxic gas [26]. Then the reduction at neutral pH of GO with ammonium thiosulphate for 20 h at 95 °C in ratio 3:1 leads to creation of the rGO/ATS flakes. The exposure to thiourea dioxide at 85 °C for 1.5 h at pH 9 of GO was used for rGO/TUD preparation with a 5:1 molar ratio. Then pressure filtration on a membrane and dialysis were used for rGO/ATS and rGO/TUD purification.

### 2.2. X-ray Photoelectron Spectroscopy of GN and rGO

The multi-chamber ultra-high vacuum (UHV) system PREVAC [PREVAC, Rogów, Poland] was used for X-ray photoelectron spectroscopy (XPS). The spectra were obtained by using a hemispherical Scienta R4000 electron analyzer [Scienta, Sweden]. Complementary equipment such as the Scienta SAX-100 X-ray source (Al Kα, 1486.6 eV, 0.8 eV band) equipped with the XM 650 X-ray Monochromator [Scienta, Sweden] (0.2 eV band) was used. The pass energy of the analyzer was set to 200 eV for survey spectra (with 500 meV steps), and 50 eV for regions (high-resolution spectra): C1s, O1s, N1s, S2p, and F1s (with 50–100 meV step). The base pressure in the analysis chamber was 5·109 mbar. During the spectra collection, it was not higher than 2 × 10^–8^ mbar.

### 2.3. Cell Culture

For cell culturing [27], the human glioblastoma U87 MG (ATCC^®^ HTB-14™) and bone marrow stromal Hs5 (ATCC^®^ CRL-11882™) cell lines were used from the American Type Culture Collection (Manassas, VA, USA). Cells were cultured in Dulbecco’s modified Eagle’s medium (DMEM) supplemented with 10% fetal bovine serum (Life Technologies, Houston, TX, USA) and 1% antibiotic-antimycotic mixture containing penicillin and streptomycin (Life Technologies, Houston, TX, USA) at 37 °C under 5% CO_2_ and 95% humidity in an INCOMED153 (Memmert GmbH & Co. KG, Schwabach, Germany).

### 2.4. Cell Viability Assay—Neutral Red

For the cell viability studies, the neutral red assay was used. A total of 2 × 10^4^ cells were cultured in 96-well plates for 24 h. Then the culture medium was replaced by another containing 10% solutions of GN and rGO flakes at the concentrations of 10, 25, 50, and 100 μg/mL. As controls, the cells incubated without flakes were used. After 24 h, 10 μL of 0.33% neutral red solution in DPBS (Thermo Fisher Scientific, Waltham, MA, USA) was added for 4 h at 37 °C. Subsequently, the culture medium with neutral red was removed, and the cells were washed with 200 µL of fixative solution (0.1% CaCl_2_ in 0.5% formaldehyde) for 3 min. The wells were then supplemented with 100 µL of solubilization solution (1% acetic acid in 50% ethanol), incubated for 10 min at room temperature, and mixed gently by pipetting. Measurements were performed at a wavelength of 540 nm using a spectrophotometer (Infinite M200, Tecan, Durham, NC, USA) and a reference 690 nm. Cell viability was expressed as a percentage compared with the control.

### 2.5. Wound Healing Assay

Cell invasion was assessed using two-well culture-inserts (No. 80209, Animalab, Germany) in 6-well plates. A total of 2 × 10^4^ cells/well cells were seeded and cultivated in two-well culture-inserts and grown until a monolayer was obtained. The growth area in the culture-insert was 0.22 cm^2^ per well. The cell-free gap width was 500  ±  100 µm. After a few days of incubation, inserts were removed and the cell layer was washed with PBS (Life Technologies, Houston, TX, USA). The plate was filled with 1.8 mL of the medium in each well. Two hundred microliters of aqueous solutions of GN and rGO flakes at a concentration of 250 µg/mL were added to the appropriate wells. Cells were cultured for 48 h and then fixed after 0, 12, 24, and 48 h with 4% paraformaldehyde and stained with May-Grünwald Giemsa stain. Photographs were taken at the same area at 2.5× magnification of the wound using an inverted light microscope. Images were analyzed using the ImageJ software (National Institutes of Health, Bethesda, MD, USA).

### 2.6. Motility Cells in Co-Culture

For the selectivity assay, we used two dyes: Cell Tracking Dye Kit (Green) for the Hs5 line (ab138891, Abcam, Cambridge, UK) and Cell Tracking Dye Kit (Orange) for the U87 line (ab138892, Abcam, Cambridge, UK). Approximately 2 × 10^5^ cells were used for labelling. Tracking dye green was diluted 1:1000 in assay buffer and 500 μL was added to 500 μL Hs5 cell suspension. Tracking dye orange was diluted 1:50 in assay buffer and 100 μL was added to 900 μL U87 cell suspension. Cells were incubated for 30 min at 37 °C. After staining, the cells were washed thrice with 20 mM HEPES buffer (HBBS). Cell pellets were suspended in 1 mL of growth medium DMEM and applied to the IBIDI dish 35 mm (Cat. No:81156) in a ratio of 1:1. A co-culture was incubated for 24 h (after the culture medium was replaced with that containing 10% solutions of the flakes of GN and rGOs at the concentrations of 25 μg/mL) and visualized on a FV10-ASW 4.2 (Olympus, Tokyo, Japan) in 200 repetitions every 5 min—Multi Area Time Lapse. The images were analyzed using two fluorescence channels for Ex/Em = 490/520, Ex/Em = 540/570, and differential interference contrast (DIC). After visualization, a film was created using the software FV3000 (Olympus, Tokyo, Japan), and the selectivity of the studied cell flakes in the co-cultures was analyzed. For more detailed analysis, films in the oib format were analyzed using the (Fiji Is Just) ImageJ program with the TrackMate overlay [28]. Cell mobility was analyzed after separating the cells into green (Hs5) and red (U87) channels. Enhanced contrast was used with a value of 0.5%, and in the LoG detector window, the value was 30,000 microns for the estimated blob diameter and for the threshold. Then, based on the values of track duration and tracking distance traveled from the track statistics window, the values of the cell motility of individual populations were calculated.

### 2.7. Total RNA Isolation and cDNA Synthesis

U87 and Hs5 cells were cultured in six-well plates in three independent replicates in the aqueous solutions of the GN and rGO flakes (25 µg/mL) for 24 h. After trypsinization, cells were centrifuged for 5 min at 400× *g* and washed twice with PBS. For the total RNA isolation, the NucleoSpin RNA Mini Kit for RNA Purification (MACHEREY-NAGEL GmbH & Co. KG Allentown, PA, USA) was used according to the manufacturer’s instructions. The RNA concentration and purification were measured using a NanoDrop OneC spectrophotometer (Thermo Scientific, Wilmington, DE, USA). The High Capacity Reverse Transcription Kit (Applied Biosystems, Foster City, CA, USA) was used for the cDNA that was synthesized. The procedure was performed under the following cycling conditions: 10 min at 25 °C, 120 min at 37 °C, and 5 min at 4 °C using a 2720 Thermal Cycler (Thermo Fisher Scientific). The cDNA concentration and purification were measured by a NanoDrop OneC spectrophotometer and stored for further analysis at −20 °C [29].

### 2.8. Gene Expression

The ΔΔCt method was used to determine mRNA expression: ΔΔCT = ΔCT test sample—ΔCT calibrator sample. The Power SYBR™ Green PCR Master Mix (Thermo Fisher Scientific) was used; 100 ng of cDNA was prepared for each reaction. Gene-specific primers (Table 1) were prepared by Genomed (Warsaw, Poland)*,* and *rpl13a* was used as the housekeeping [29,30].

### 2.9. Immunoblotting—Protein Expression

Immunoblotting was used to evaluate integrin α5, integrin β1, N-cadherin, β-catenin, and focal adhesion kinase (FAK) expression. The cells were cultured as described in the section “Cell cultures”. Ice-cold lysis buffer TX100 (150 mM NaCl, 1% Triton X-100, 50 mM TRIS pH 8) supplemented with protease (Sigma-Aldrich, St. Louis, MO, USA) at a ratio of 100:1 (TX100: protease) was used for whole-cell protein extracts isolation. Cells were centrifuged for 30 min at 12,000× *g* at 4 °C. The protein concentration in supernatant was measured using a Bicinchoninic Acid Kit (Sigma-Aldrich, St. Louis, MO, USA). Proteins were denatured for 5 min using the β-mercaptoethanol (Bio-Rad Laboratories, Munich, Germany). Equal amounts of protein from each sample were loaded onto a 7.5% polyacrylamide gel. Electrophoresis was performed at 100 mA and 120 V for 1.5 h in 25 mM TGS [Tris-glycine-sodium dodecyl sulfate (SDS) buffer]. Then the Trans-Blot Turbo Transfer System (Bio-Rad Laboratories, Munich, Germany) was used for protein transfer to polyvinylidene difluoride (PVDF) membranes and blocked with I-BLOCK^TM^ reagent (0.2%) (Applied Biosystems, Bedford, MA, USA) in PBS with Tween-20 (0.1%) for 60 min. The primary antibodies such as integrin α5 monoclonal antibody (ab150361, Cambridge, UK), integrin β1 monoclonal antibody (ab179471, Cambridge, UK), N-cadherin monoclonal antibody (MA1-159, Thermo Fisher Scientific, Waltham, MA, USA), β-catenin polyclonal antibody (PA5-19469, Thermo Fisher Scientific, Waltham, MA, USA), and FAK monoclonal antibody (ab40794, Cambridge, UK) were incubated with the membranes overnight at 4 °C. Then they were washed in PBS with Tween-20 (0.1%) and incubated with the diluted secondary antibodies such as goat anti-mouse IgG H&L (AP, ab97020 Cambridge, UK) and goat anti-rabbit IgG H&L (AP, ab97048, Cambridge, UK) for 1 h. The Novex™ AP chemiluminescent substrate (CDP-Star™) (WP20002, Thermo Fisher Scientific, Waltham, MA, USA) was used for protein detection and then visualized using Azure c400 (Azure Biosystems, Dublin, CA, USA). For protein normalization, the glyceraldehyde-3-phosphate dehydrogenase (GAPDH, MA5-15738, Thermo Fisher Scientific, Waltham, MA, USA) was used as the loading control. The background corrections were performed using ImageJ^®^ 1.48v (National Institutes of Health, Bethesda, MD, USA) [23].

### 2.10. Statistical Analysis

GraphPad Prism 8.4.3 (GraphPad Software Inc., La Jolla, CA, USA) was used for the data analysis. Differences between the groups were tested using Bonferroni’s tests and Dunnett’s multiple comparison tests. All mean values are presented as standard deviations or standard errors. Differences were considered statistically significant at *p* < 0.05.

## 3. Results

### 3.1. X-ray Photoelectron Spectroscopy of GN and rGO

**Figure 1:**
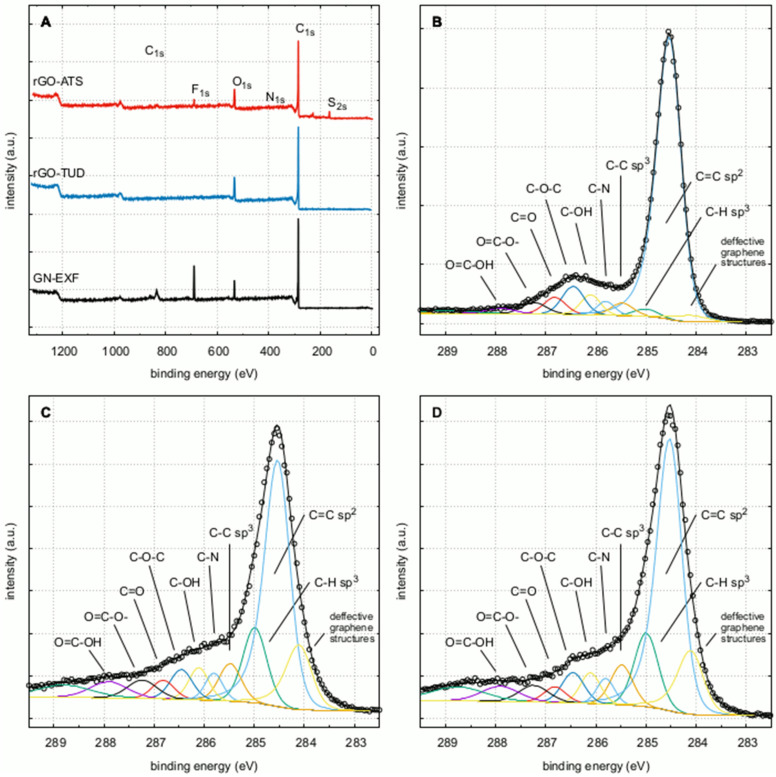
X-ray photoelectron spectroscopy (XPS) results: survey spectra of GN/ExF, rGO/TUD, and rGO/ATS (**A**), C1s high-resolution spectra (HR) of GN-EXF (**B**), rGO-TUD (**C**), and rGO-ATS (**D**). Abbreviations: rGO, reduced graphene oxide; GN, graphene; C, control group (untreated group); ExF, exfoliation; ATS, ammonium thiosulphate; TUD, thiourea dioxide.

**Figure A2:**
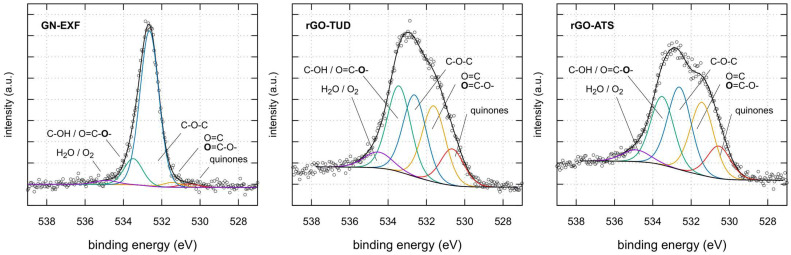
XPS results: O1s high resolution spectra of GN/ExF, rGO/TUD and rGO/ATS.

The XPS survey spectra of the samples are shown in Figure 1A. Carbon and oxygen were the major contributors to the surface composition (81.5 and 85.3 atomic%, respectively). The presence of nitrogen (rGO/TUD and rGO/ATS) and sulfur in the rGO/ATS sample was due to the different substrates and preparation methods employed. The presence of fluorine in the GN/EXF and rGO/ATS samples was caused by the use of Capstone FS-30 during the preparation procedure. The high-resolution spectra of the C1s region are presented in Figure 1B–D. The peak model for this region was based on the works of Koinuma [35], Morais [36], Rabchinskii [37], Radaelli [38], and Barinov [39]. The GN/ExF spectrum featured a well-developed narrow (FWHM = 0.56 eV) peak at 284.5 eV, corresponding to aromatic sp2 carbon. Defective graphene structures were visible at 284.1 eV, and aliphatic moieties C-C sp3 and C-H sp3 were visible at 285.0 and 285.5 eV, respectively [35,39,40]. The surface of GN/ExF flakes was partially oxidized. Epoxy groups were the most abundant ones (5.7 atomic%). Other oxygen species such as hydroxyl, carbonyl, and carboxyl groups, were also present and reach 12.6% at. in total [35,36,38]. The results are in line with literature data [35,36,41]. The reduction of GO using ammonium thiosulfate (rGO/ATS) and thiourea dioxide (rGO/TUD) leads to the production of more defective forms of graphene. The HR spectra of rGO/ATS and rGO/TUD are shown in Figure 1C,D, respectively. The main sp2 carbon peaks are well developed, but less pronounced (46.7 and 46.6% at., respectively). Compared to sample GN/ExF, peaks at ~284.1 eV and ~283.5 eV, corresponding to defective structures [39,42], are higher: 13.3% at. (rGO/ATS) and 12.6% at. (rGO/TUD) compared to 2.3% at. High resolution spectra of O1s region are presented in Figure A2. The spectra were fitted with five peaks, corresponding to the following moieties: quinones (~530.6 eV), carbonyl oxygen (~531.5), epoxy (~532.6), aromatic hydroxyl and carboxyl groups (~533.4) and adsorbed oxygen species (H2O/O2) [39,43,44,45]. On the GN/ExF surface, the C-O-C epoxy groups were most abundant (80% of all the oxygen species); however, it should be kept in mind that the total oxygen amount in this sample was the lowest: 6 % at., compared to 11.5% at. for rGO-ATS and 13.3% at. for rGO/TUD. The reduced graphene oxides (rGO/ATS and rGO/TUD) contained considerable amounts of other oxygen species. Most notably, the amounts of carbonyl oxygen were more than tenfold higher (27.1% at. for rGO/ATS and 25.4% at. for rGO/TUD) compared to GN/ExF (2.5 % at.). The amounts of hydroxyl oxygen were almost two-fold higher (25.1% at. for rGO/ATS and 28.6% at. for rGO/TUD) versus the exfoliated graphene (13.3% at. for GN/ExF) and the amounts of quinone oxygen were more than sevenfold higher (12.1% at. for rGO/ATS and 12.4% at. for rGO/TUD vs. 1.7% at. for GN/ExF). GN/ExF and rGO/TUD samples contained tiny amounts of sulphur–less than 0.2% at. On the contrary, the rGO/ATS sample contained more than 4% at. of this element, due to non-ideal removal of the reducing agent (ammonium tiosulphate). The reduced graphene oxide samples contained less than 1.5% at. of nitrogen. The high-resolution spectra show that its main forms were pyridinic nitrogen at ~398.2 eV, amines at ~399.9 eV, and quarternary nitrogen [40,46,47].

### 3.2. Cell Viability Assay—Neutral Red

**Figure 2:**
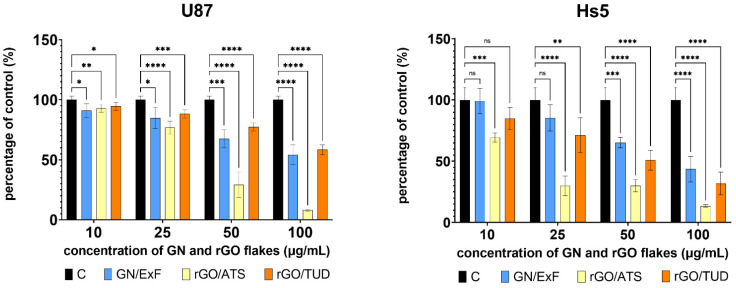
Cell viability of U87 and Hs5 cells treated and untreated with GN and rGO flakes, as evaluated by the neutral red assay. U87 and Hs5 cells were exposed to GN and rGO flakes at concentrations of 10, 25, 50, and 100 μg/mL for 24 h. Values are expressed as mean ± standard deviation. Statistical significance between the control and treated cells is indicated by an asterisk and was assessed using Bonferroni’s multiple comparison test. Differences with *p* < 0.05 were considered statistically significant. One asterisk (*) indicates *p* < 0.01, two asterisk (**) indicates *p* < 0.005, three asterisks (***) indicate *p* < 0.001, four asterisk (****) indicate *p* < 0.0001. Abbreviations: rGO, reduced graphene oxide; GN, graphene; C, control group (untreated group); ExF, exfoliation; ATS, ammonium thiosulphate; TUD, thiourea dioxide; ns, not significant.

First, we assessed the effect of GN and rGO flakes on the viability and cytotoxicity of glioblastoma multiforme (U87) and normal (Hs5) cell lines using a neutral red assay (Figure 2). In the case of neoplastic cells of the U87 line in all groups treated with GN and rGO flakes, statistically significant changes in viability were observed at all concentrations tested. In healthy Hs5 cells, the most statistically significant changes were observed after treating the cells with rGO/ATS and rGO/TUD flakes at 25, 50, and 100 μg/mL. In the U87 cell line, a highly statistically significant (*p* < 0.0001) reduction in viability was observed in the groups treated with rGO/ATS flakes at concentrations of 50 μg/mL (29.16% ± 2.54) and 100 μg/mL (7.98% ± 1.25) and in the groups treated with rGO/TUD flakes at a concentration of 100 μg/mL (58.31% ± 2.06). In healthy Hs5 cells, the most significant reduction in viability (*p* < 0.0001) was observed in the groups treated with rGO/ATS at 25 μg/mL (29.95% ± 5.27), 50 μg/mL (29, 98% ± 4.58), and 100 μg/mL (13.55% ± 4.13). Interestingly, in the groups treated with 100 μg/mL rGO/TUD, a greater reduction in viability was observed in healthy Hs5 cells(31.8% ± 5.6), compared to the U87 tumor cells. Based on the results of the neutral red test, the optimal concentration of 25 µg/mL GN and rGO flakes was selected for further experiments.

### 3.3. Wound Healing Assay

**Figure 3:**
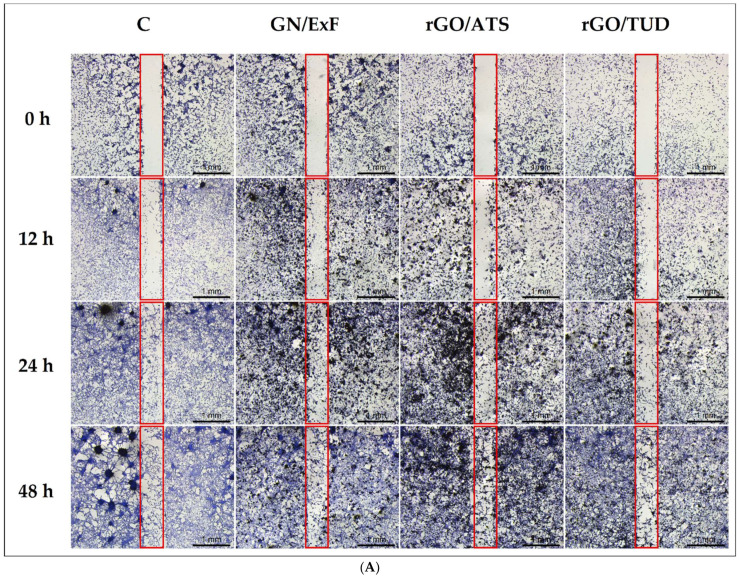

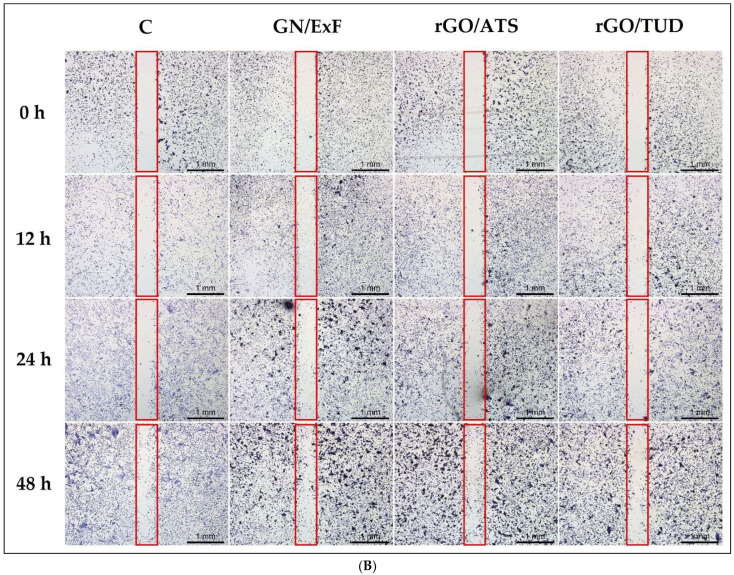
Analysis of U87 (**A**) and Hs5 (**B**) cell invasion and migration after 0, 12, 24 and 48 h of cultivation. U87 and Hs5 cells were exposed to GN and rGO flakes at a concentration of 25 μg/mL. Notes: For better contrast, cells were fixed and stained with the May Grünwald–Giemsa method. The scratch area is marked with a red frame. Scale bar: 1 mm. Abbreviations: rGO, reduced graphene oxide; GN, graphene; C, control group (untreated group); ExF, exfoliation; ATS, ammonium thiosulphate; TUD, thiourea dioxide.

A wound healing assay (Figure 3) was performed to evaluate the migration and proliferation of the U87 and Hs5 cells treated with the GN and rGO flakes. U87 cells (Figure 3A) migrated faster than healthy Hs5 cells (Figure 3B) and completely covered the free space of the scratch after 24 h. However, Hs5 cells did not completely cover the scratch even after 48 h. The results of the wound healing assay indicate that the treatment with rGO/TUD flakes reduced the migration of U87 cells compared to the control cells. Moreover, in the case of U87 cells, a decrease in spheroid formation was observed, especially after 48 h of incubation in the groups treated with rGO flakes compared to the control cells. In healthy Hs5 cells, reduced cell migration was observed after treatment with rGO/TUD flakes for 48 h.

### 3.4. Motility

**Figure 4:**
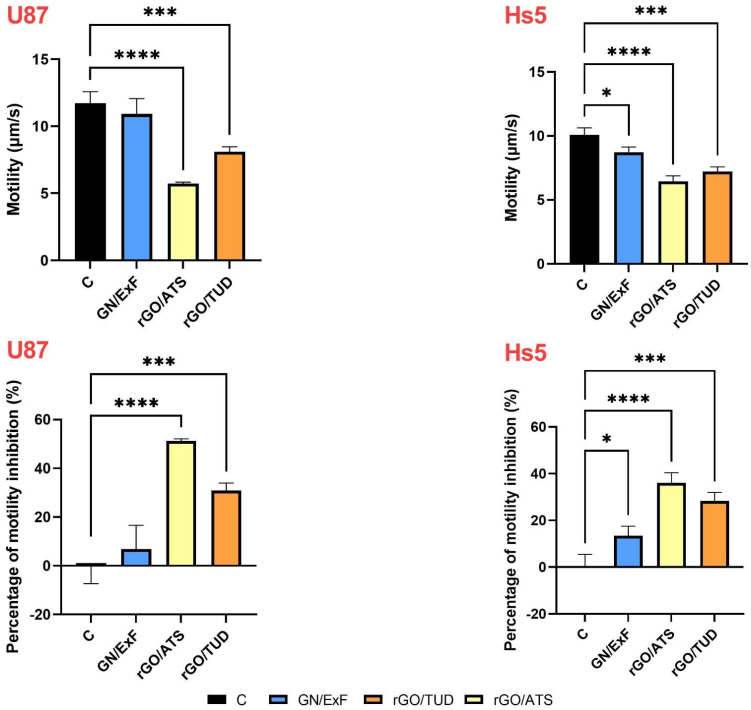
Analysis of U87 and Hs5 cell motility (μm/s) and percentage of motility inhibition (%) in co-culture treated with graphene (GN) and reduced graphene oxide (rGO). Statistical significance between the control and treated cells is indicated by an asterisk and was assessed using Bonferroni’s multiple comparisons test. Differences with *p* < 0.05 were considered statistically significant. One asterisk (*) indicates *p* < 0.05, three asterisk (***) indicates *p* < 0.001 and four asterisk (****) indicate *p* < 0.0001. Abbreviations: rGO, reduced graphene oxide; GN, graphene; C, control group (untreated group); ExF, exfoliation; ATS, ammonium thiosulfate; TUD, thiourea dioxide. Notes: Cell mobility data were analyzed from three independent cultures. Within each co-culture, 100 cell pathways were analyzed in ImageJ using the TrackMate overlay. The average track duration and distance traveled were obtained from the 100 paths. Three independent means from 100 paths were used for statistical calculations.

Next, we analyzed the motility (Figure 4, Table 2, Table 3) of U87 glioma tumor cells and healthy Hs5 cells in co-cultures after treatment with GN/ExF graphene flakes and rGO/ATS and rGO/TUD. First, based on the obtained results, it can be observed that the speed of tumor (U87: 11.73 μm/s) and healthy (Hs5: 10.08 μm/s) cells differed in the control co-cultures. U87 cells were found to be faster than Hs5 cells, and their mobility decreased significantly by 51.2% (*p* < 0.0001) and 30.86% (*p* < 0.01) upon treatment with rGO/ATS and rGO/TUD flakes, respectively. In the case of healthy Hs5 cells, mobility decreased significantly by 13.41% (*p* < 0.05), 36.06% (*p* < 0.0001), and 28.36% (*p* = 0.0001) after treatment with GN/ExF, rGO/ATS, and rGO/TUD, respectively, compared to control cells.

### 3.5. Gene Expression

**Figure 5:**
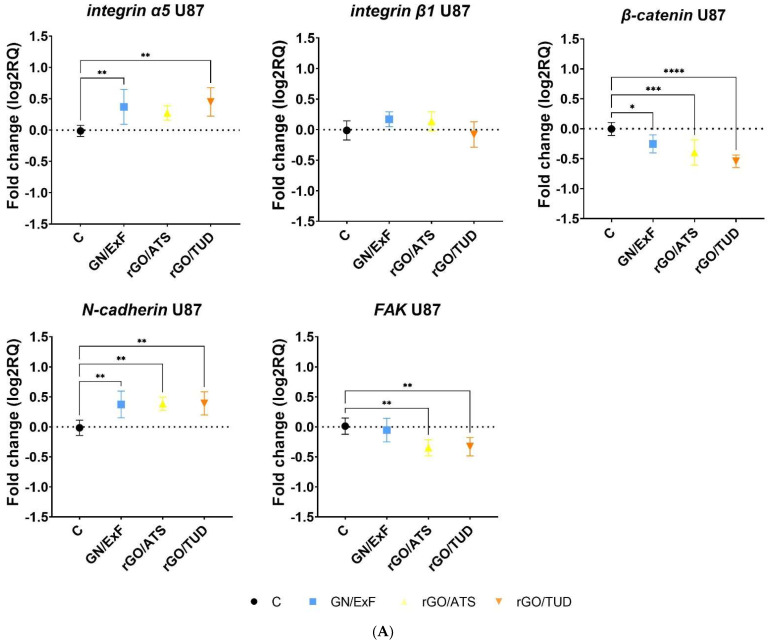

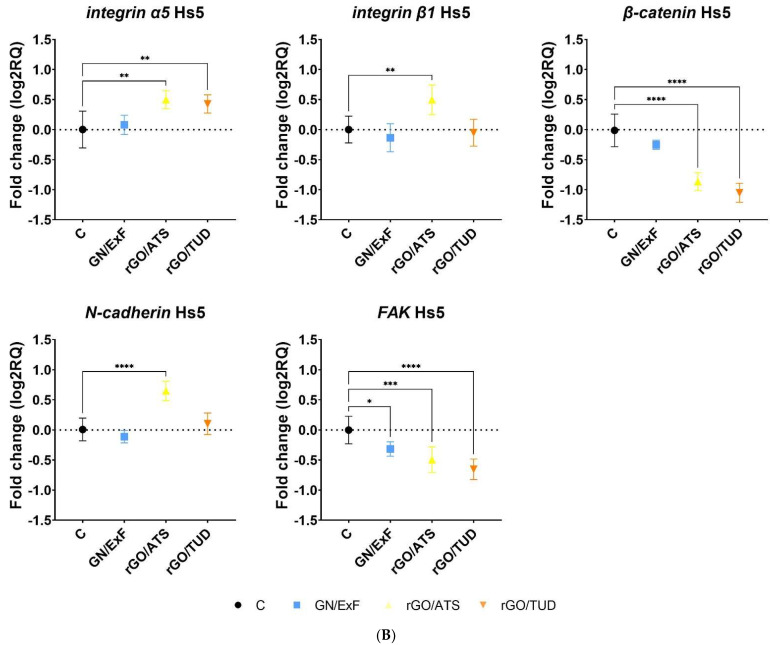
Analysis of the mRNA expression levels of the integrin α5, integrin β1, β-catenin, N-cadherin, and FAK genes after GN and rGO treatment at 25 µg/mL concentration for 24 h in U87 (**A**) and Hs5 (**B**) cells. The results are calculated relative to the control values. Log2RQ (log2 relative quantitation) values for all genes are normalized to the housekeeping gene RPL13A. Statistical significance between the control and the treated cells is indicated by an asterisk and was assessed using Bonferroni’s multiple comparisons test. Differences with *p* < 0.05 were considered statistically significant. One asterisk (*) indicates *p* < 0.05, two asterisk (**) indicates *p* < 0.01, three asterisk (***) indicates *p* < 0.001 and four asterisks (****) indicate *p* < 0.0001. Abbreviations: rGO, reduced graphene oxide; GN, graphene; C, control group (untreated group); ExF, exfoliation; Term, thermal; ATS, ammonium thiosulfate; TUD, thiourea dioxide; rpl13a, ribosomal protein L13a; log2RQ, log2 relative quantitation.

The next stage of this study was to evaluate the influence of the studied forms of GN and rGO on the expression of the selected adhesion and cell migration markers in U87 glioma cells and healthy Hs5 cells (Figure 5). A statistically significant increase in integrin α5 expression was observed in U87 glioma cells (Figure 5A) after treatment with GN/ExF (log2RQ = 0.38 ± 0.1121) and rGO/TUD (log2RQ = 0.46 ± 0.1121) and in healthy Hs5 cells (Figure 5B) after treatment with rGO/ATS (log2RQ = 0.49 ± 0.1173) and rGO/TUD (log2RQ = 0.43 ± 0.1173). Compared with U87 glioma cells, no change in integrin α5 expression was observed in Hs5 cells after treatment with GN/ExF flakes. Subsequently, the expression analysis of integrin β1 revealed no statistically significant changes in its expression in U87 glioma cells treated with GN and rGO graphene flakes. However, a statistically significant increase in integrin β1 expression was observed in Hs5 cells treated with rGO/ATS (log2RQ = 0.49 ± 0.1335). Next, β-catenin expression was analyzed after treatment with GN/ExF flakes (log2RQ = −0.25 ± 0.08658), rGO/ATS (log2RQ = −0.39 ± 0.08658) and rGO/TUD (log2RQ = −0.54 ± 0.08658). A statistically significant reduction in β-catenin expression was observed in U87 glioma cells, with the most significant reduction in cells treated with rGO/TUD flakes (log2RQ = −0.54 ± 0.08658). In healthy Hs5 cells treated with GN/ExF flakes, the expression of β-catenin decreased, but this was not a statistically significant change. However, a greater reduction in β-catenin expression was observed in Hs5 cells treated with rGO/ATS (log2RQ = −0.85 ± 0.1025) and rGO/TUD (log2RQ = −1.04 ± 0.1025), compared with that in U87 glioma cells. Further, a statistically significant increase in N-cadherin expression was observed in U87 glioma cells (*p* < 0.01) after treatment with GN/ExF (log2RQ = 0.39 ± 0.09812), rGO/ATS (log2RQ = 0.4 ± 0.09812), and rGO/TUD (log2RQ = 0.41 ± 0.09812), whereas in healthy Hs5 cells (*p* < 0.0001), it was observed only after rGO/ATS treatment (log2RQ = 0.64 ± 0.09322). Expression analysis of FAK showed a statistically significant (*p* < 0.01) reduction in its expression in U87 glioma cells after treatment with rGO/ATS (log2RQ = −0.36 ± 0.09078) and rGO/TUD (log2RQ = −0.34 ± 0.09078), whereas in healthy Hs5 cells, it was observed after treatment with all tested flakes: GN/ExF (log2RQ = −0.31 ± 0.1084), rGO/ATS (log2RQ = −0.49 ± 0.1084), and rGO/TUD (log2RQ = −0.65 ± 0.1084).

### 3.6. Protein Expression

**Figure 6:**
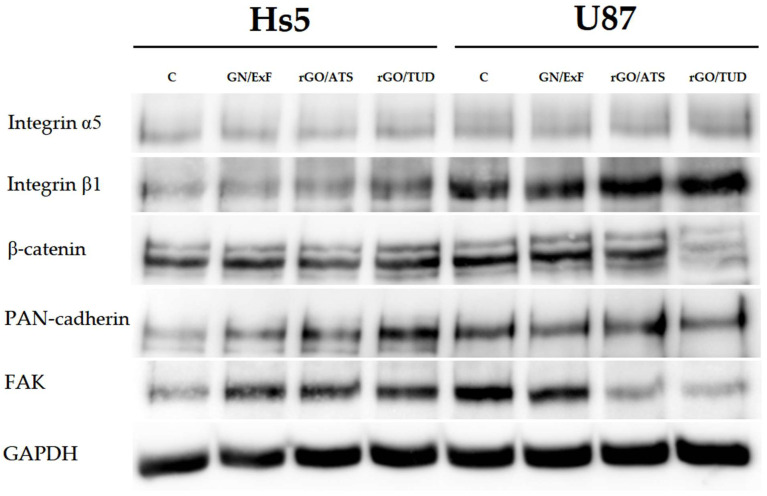
Western blot analysis of integrin α5, integrin β1, β-catenin, PAN-cadherin, and FAK after GN and rGO treatment at 25 µg/mL concentration for 24 h in U87 and Hs5 cells. GAPDH was used as a loading control. Abbreviations: rGO, reduced graphene oxide; GN, graphene; C, control group (untreated group); ExF, exfoliation; term, thermal; ATS, ammonium thiosulfate; TUD, thiourea dioxide; GAPDH, glyceraldehyde 3-phosphate dehydrogenase.

Changes in expression at the protein level were confirmed using Western blotting. Expression levels of proteins involved in cell adhesion and migration (integrin α5, integrin β1, β-catenin, PAN-cadherin, and FAK) were analyzed in U87 glioma cells and compared with the corresponding protein levels in healthy Hs5 cells treated with different graphene flakes (GN and rGO) (Figure 6). No significant changes in integrin α5 expression were observed in Hs5 cells treated with GN and rGO flakes. In U87 glioma cells, only a slight increase in integrin α5 expression was observed after treatment with rGO/TUD flakes. In healthy Hs5 cells, a slight increase in integrin β1 expression was observed after treatment with GN/ExF and rGO/ATS flakes, but the greatest increase was observed after treatment with rGO/TUD flakes. In U87 glioma cells, we observed an increase in integrin β1 expression after treatment with rGO/ATS and rGO/TUD flakes. In normal Hs5 cells, no change in β-catenin expression was observed upon treatment with all tested flakes. In U87 glioma cells, a slight decrease in β-catenin expression was observed after treatment with rGO/ATS flakes, while a strong decrease was observed after treatment with rGO/TUD flakes. PAN-cadherin expression increased in healthy Hs5 cells treated with GN/ExF and rGO/ATS flakes; however, the greatest increase was observed in cells treated with rGO/TUD flakes. In U87 glioma cells, no changes in PAN-cadherin expression were observed. A significant increase in FAK expression was observed in Hs5 cells after treatment with all tested GN/ExF, rGO/ATS, and rGO/TUD flakes. However, U87 glioma cells had the opposite effect, with the group treated with GN/ExF flakes showing a decrease in FAK expression and the groups treated with rGO/ATS and rGO/TUD flakes showing a much greater reduction in FAK expression.

## 4. Discussion

In the present investigation, we hypothesized that the cytotoxicity of rGOs may result mainly from the direct contact of their surface functional groups with the GBM cell membrane and may lead to the disruption (activation/inhibition) of signaling pathways in the plasma membrane and inside the cell, thus modifying the mRNA and protein expression of molecules related to migration and adhesion. The study of the interaction between the GN materials and the expression of selected proteins was thought to reveal the mechanisms underlying the cytotoxicity of these materials in the cell membrane and their innovative application potential in therapy. Therefore, the present study focused on elucidating the migration and adhesion mechanisms of U87 GMB cells treated with GN and rGO flakes in comparison to normal Hs5 cells.

During a first stage of this study, the cytotoxicity of GN and rGO flakes was analyzed using the neutral red test (Figure 2). Having regard to the fact that living cells incorporate weakly cationic dyes into lysosomes, which first cross the cell membranes by non-ionic passive diffusion compared to damaged or dead cells [48], the dose-dependent cytotoxicity of GN and rGO can be determined by the neutral red uptake assay. Neutral red analysis showed the cytotoxicity of the tested GN materials against U87 glioma tumor cells and Hs5 healthy cells (Figure 2). RGO flakes were more toxic to both lines than GN flakes. Particular differences in the sensitivity of cells to the tested materials were observed in the case of the rGO/ATS flakes at a concentration of 25 µg/mL. In U87 glioma cells, viability was 76.76%, while in healthy Hs5 cells, viability after treatment decreased to 29.95%. Another significant difference in the sensitivity of the tested materials was observed in the case of the rGO/TUD flakes at a concentration of 100 µg/mL. In U87 glioma cells, we observed a viability of 58.31%, while in the case of healthy Hs5 cells, the viability decreased to 31.81%, indicating the greater toxicity of the examined flakes against healthy cells. In our previous studies, Szczepaniak et al. used the MTT metabolic activity test in healthy Hs5 cells and showed a smaller decrease in metabolic activity than in U87 tumor cells after treatment with the same materials [49]. For example, in the group treated with rGO/TUD flakes at a concentration of 100 µg/mL, the U87 glioma cells showed a decrease in viability of 8.69 ± 12.88%, while in the case of healthy Hs5 cells at the same concentration, the decrease in viability was approximately 65%. Moreover, in the case of the treatment with rGO/ATS flakes, a 57.03% viability was observed at a concentration of 100 µg/mL in U87 glioma cells, whereas in healthy Hs5 cells at the same concentration, the decrease in viability was approximately 65% [49]. Neutral red uptake depends on the ability of the cell to maintain a pH gradient through ATP production. At a physiological pH, the dye shows a charge close to zero, allowing it to penetrate the cell membranes. In contrast, a proton gradient at the center of lysosomes maintains a its pH lower than that of the cytoplasm. In this way, the dye becomes charged inside the lysosomes [48]. Therefore, the differences in the cytotoxicity results of the presented neutral red and MTT assays may be caused, among others, by GN and rGO flakes blocking the non-ionic diffusion of passive neutral red through the cell membrane, and the flakes can also disrupt the proton gradient, keeping the pH of lysosomes lower than that of the cytoplasm. Our MTT and neutral red assay results are consistent with those of previous studies, which indicate that GN and rGO flakes show significant toxicity against several glioblastoma multiforme cell lines, among others, U251, U87, and U118 [50,51,52,53]. Muthoosamy et al. observed a significant difference in the viability of U87 cells, starting from a concentration of 25 µg/mL (*p* < 0.01) for GO and rGO; GO had an IC50 of 26.27 μg/mL, which turned out to be more toxic to U87 cells than rGO, with an IC50 of 132.40 μg/mL). They also showed that rGO showed no toxicity against normal cell lines, with cell viability above 90%, even at a concentration of 50 µg/mL [54].

Next, we performed a wound healing assay to assess the migration of U87 and Hs5 cells after treatment with GN and rGO flakes (Figure 3). We observed that U87 cells migrated faster than normal Hs5 cells and almost completely covered the free gap after 24 h, which is consistent with the observations of Formolo et al., who showed that the level of invasiveness of the U87 glioma line was 29 times higher than that of the T98G, LN18, and U118 cell lines [55]. Treatment of cells with rGO/TUD flakes reduced the migration of U87 glioma cells, leading to slower crack growth. Hs5 cells showed low mobility, and additional treatment with the tested flakes did not change mobility in relation to the control group. Cancer cells migrate by gradually degrading their surrounding extracellular matrix (ECM) to create their own migration tracks by following ‘leader’ cancer cells or cancer-associated stromal cells that open up paths for migration or by moving through pre-existing channel-like tracks created by anatomical structures [56]. Xiao et al. observed that U87 cells in co-cultivation conditions with healthy neurons and glium had significantly higher mobility [57]. Wierzbicki et al. also observed that treatment with NG and nGO nanoparticles at a concentration of 50 μg/mL reduced the two-dimensional migration of U87 and U118 GBM cells [25]. These results were confirmed by the mobility analysis of the co-culture of U87 tumor cells and healthy Hs5 cells (Figure 4). It was observed that the materials with the strongest properties that inhibited the mobility of U87 glioma cells were rGO/ATS flakes (difference between the control (11.73 µm/s) and rGO/ATS-treated cells (5.72 µm/s) was 6.01 µm/s) followed by rGO/TUD flakes (difference between the control (11.73 µm/s) and rGO/TUD-treated cells (8.11 µm/s) was 3.62 µm/s). In normal Hs5 cells, the treatment with rGO/ATS and rGO/TUD flakes reduced the mobility to 3.64 µm/s and 2.86 µm/s, respectively, which was lower than that of the corresponding treated U87 glioma cells. Overall, the flakes tested inhibited the mobility of both normal and tumor cells; however, for tumor cells that naturally have higher mobility, this change was stronger.

The factors that regulate cell mobility are cell adhesion molecules and ECM receptors [58]. These include the integrins α5 and β1, β-catenin, N-cadherin, PAN-cadherin, and FAK, which were analyzed in the present study. Integrins are the principal ECM receptors in animal cells. Integrin α5β1 is a receptor for fibronectin (a glycoprotein found in the extracellular matrix) that plays a very important role in tumor progression, metastasis, and/or resistance to therapy [59,60,61]. Integrin α5β1 is expressed at a much higher level in GBM cells than in normal brain tissue, suggesting it potential role in GBM development or progression [62]. The results of our study showed a statistically significant increase in the expression of integrin α5 mRNA levels after treatment with GN/ExF flakes (log2RQ = 0.38 ± 0.1121) and rGO/TUD (log2RQ = 0.46 ± 0.1121) in the U87 cell line. No statistically significant changes were observed in the expression of integrin βl mRNA upon treating U87 cells with GN flakes. In contrast, in healthy Hs5 cells, the expression of integrin β1 mRNA increased significantly in the group treated with rGO/ATS flakes (log2RQ = 0.49 ± 0.1335). Analysis of integrin α5 expression at the protein level showed no difference after treatment with GN flakes. Integrin βl protein expression increased when U87 glioma cells were treated with rGO/ATS and rGO/TUD flakes. Moreover, the expression of integrin β1 was much higher in U87 cells than in healthy Hs5 cells. Janouskova et al. showed that high expression of integrin α5β1 is associated with worse prognosis in patients with GBM [63]. Thus, an increase in integrin α5 expression after treatment with GN/ExF and rGO/TUD flakes may be detrimental to invasiveness and patient survival. Riemenschneider et al. observed integrin α5β1 overexpression at the protein level in a significant percentage of human glioblastoma biopsies [64]. Moreover, Janouskova et al. showed that p53 activation by nutline-3a inhibits the expression of integrin α5β1 [63]. Therefore, we suspect that the expression of integrin β1 at the mRNA level after treatment with GN flakes did not change in U87 glioma cells because of the activation of p53, which inhibits the expression of integrin β1. It is likely that the GN flakes act on the signaling pathway, similar to nutlin-3a. Moreover, ECM components, such as vitronectin, collagen, and fibronectin, are natural integrin ligands. Each pair of ab integrins contains a specific set of ECM proteins [65]. Therefore, GN flakes attached to the ECM can actively block access to ligands of integrin β1, such as fibronectin, and by binding to the GBM cell membrane, actively blocking integrin β1 itself and indirectly increasing the expression of integrin β1 at the protein level. Thus, after flake treatment, tumor cells showed no alteration in the expression of integrin βl at the mRNA level, but showed increased expression at the protein level after rGO/ATS and rGO/TUD treatment. This may be due to the fact that tumor cells have a stock of integrin β1 mRNA and use it under unfavorable conditions to increase integrin β1 expression at the protein level, compensating for the stress of blocking fibronectin ligands in the ECM and integrin β1 itself, thereby increasing cell–ECM interaction and reducing migration.

β-catenin is a key component of the canonical Wnt/β-catenin pathway. It accumulates in the nucleus, where it interacts with transcriptional co-regulators, including Tcf-4 and Lef-1, to form the B-catenin/Lef/Tcf complex, which regulates the transcription of several genes involved in proliferation, differentiation, survival, and apoptosis [66]. It appears that β-catenin expression is higher in glioma cells than in healthy brain cells. Silencing of β-catenin by siRNA in human GBM cells inhibits cell proliferation and invasiveness and induces apoptotic death [66]. Abnormal activation of the Wnt/β-catenin signaling pathway is a hallmark of many cancers [67] and may contribute to the formation and maintenance of stem cell-like phenotypes [68]. The results of our analysis show a statistically significant decrease in β-catenin mRNA expression after treatment with GN/ExF flakes (log2RQ = −0.25 ± 0.08658), rGO/ATS (log2RQ = −0.39 ± 0.08658), and rGO/TUD (log2RQ = −0.54 ± 0.08658) in U87 glioma cells. The greatest decrease in β-catenin expression after treatment with rGO/TUD flakes was confirmed by Western blot analysis in U87 glioma cells. Wierzbicki et al. also observed a reduction in the expression of cytoplasmic β-catenin at the protein level after treatment with NG and nGO nanoparticles in U118 glioma cells [25]. The reduction in β-catenin expression due to the promotion of proliferation and stem cell renewal may contribute to lowering the invasiveness of neoplastic cells, making them more sensitive to other therapies, including radiotherapy, and reducing tumor relapse after resection [69].

β-catenin, a component of the cell adhesion complex, binds to cadherin and thus regulates cell–cell adhesion. Changing the binding of β-catenin to cadherin reduces cell adhesion while promoting cell migration and epithelial-mesenchymal transition [70]. In the present study, the mRNA and protein expression levels of N-cadherin and PAN-cadherin (an antibody reactive with all types of cadherin proteins, including N-cadherin, E-cadherin, P-cadherin, and R-cadherin) were analyzed. The results of our mRNA analyses show a statistically significant increase in N-cadherin expression after treatment with all tested GN/ExF flakes (log2RQ = 0.39 ± 0.09812), rGO/ATS (log2RQ = 0.4 ± 0.09812), and rGO/TUD (log2RQ = 0.41 ± 0.09812). A feedback phenomenon probably occurred here because a slight decrease in PAN-cadherin expression at the protein level was observed after treatment with GN/ExF flakes, but no visible changes were observed after treatment with rGO/ATS and rGO/TUD flakes in U87 glioma cells. Wierzbicki et al. also observed a decrease in PAN-cadherin expression at the protein level after nGO treatment in U118 glioma cells. For the U87 lineage, no changes in expression were observed for both N-cadherin and PAN-cadherin. Wierzbicki et al. suggested that nanoparticles may change cell migration by hindering interaction between cells and ECM [25]. As the level of cadherins did not change, it should be assumed that the reduction in mobility of glioma cells and fibroblasts occurred because the proposed mechanism of changes in intercellular connections did not occur.

Focal adhesion kinase (FAK) is a multifunctional regulator of cell signaling in the tumor microenvironment [71]. During the development of various types of cancer, FAK promotes cell mobility, survival, and proliferation through kinase-dependent and independent mechanisms. In addition, pharmacological inhibition of FAK activity increases the strength of cell–cell adhesion, which may prevent cancer cells from spreading more rapidly [72]. Our mRNA-level expression analyses show a statistically significant decrease in expression following treatment with rGO/ATS (log2RQ = −0.36 ± 0.09078) and rGO/TUD (log2RQ = −0.34 ± 0.09078) flakes. Moreover, analysis of FAK expression at the protein level clearly confirmed and showed a decrease in FAK protein expression after treatment with rGO/ATS and rGO/TUD flakes in U87 glioma cells. In contrast, in Hs5 cells, an increase in FAK protein expression was observed after treatment with all GN/ExF, rGO/ATS, and rGO/TUD flakes. Lipinski et al. found that increased FAK activity was correlated with high proliferation and low migration rates in four different human GBM cell lines, suggesting that FAK is an important signaling effector in gliomas, and its regulation may be a determinant of proliferative development or migratory phenotype [73]. Thus, the reduction in the expression of FAK in U87 cells may be the reason for the decreased proliferation of U87 glioma cells after treatment with rGO/ATS and rGO/TUD flakes. The mechanism of interaction with normal cells might be different, as a decrease in FAK expression at the mRNA level and an increase in FAK expression at the protein level, with a simultaneous decrease in mobility, were observed. Canel et al. observed that alternative splicing or increased FAK mRNA expression does not always translate to increased protein levels [74]. Therefore, in normal Hs5 cells, we observed no relationship between FAK expression at the mRNA and protein levels. Moreover, FAK is involved in the formation of integrin receptor clusters upon binding of cells to ECM proteins, which may include FAK dimerization [75]. The interaction between the C-terminal domain of FAK and integrins in focal adhesion (FA) is mediated by integrin/FAK binding because the overexpression of the C-terminal fragment of FAK blocks integrin-mediated activation of FAK [76]. Therefore, we suppose that in U87 glioblastoma cells treated with rGO flakes, the reduction in FAK expression at both mRNA and protein levels may be caused by the rGO-induced increase in the expression of integrin β1.

Analyzing the results in terms of the differences between the tested materials, it is evident that rGO/ATS causes a strong decrease in viability, mobility, and mRNA expression of β-catenin and FAK, as well as an increase in the expression of N-cadherin mRNA in U87 cells. At the protein level, after treatment with rGO/ATS flakes, an increase in the expression of integrin βl and a decrease in the expression of FAK in U87 cells was observed. In contrast, rGO/TUD caused a decrease in viability in U87 glioblastoma cells, but lower than in the case of the aforementioned flakes, as well as inhibition of migration, reduced mobility, an increase in mRNA expression of integrin α5 and N-cadherin, and a decrease in the extrusion pressure of β-catenin and FAK. After treatment with rGO/TUD flakes, an increase in the expression of integrin βl and a decrease in the expression of β-catenin and FAK were observed at the protein level. For comparison, GN/ExF flakes in U87 glioma cells caused a lower cytotoxic effect, did not inhibit migration, decreased cell mobility to a lesser extent, and, at the mRNA level, increased integrin α5 and N-cadherin expression, and decreased β-catenin expression. At the protein level, there was a slight reduction in the expression of PAN-cadherin and FAK. These differences probably result from the structure of these flakes (first, the examined flakes differed in the presence of oxygen-containing surface functional groups as well as in a number of physicochemical properties). It seems that flakes with more oxygen-containing functional groups have relatively high potential and hydrophilicity, which is directly related to the size of the GN flakes that can more easily adhere to the cell wall.

## 5. Conclusions

This study expands our knowledge about the functional mechanism of carbon flakes. We showed that they can affect cell mobility by interacting with cell membrane receptors, such as integrins α5 and β1, β-catenin, cadherins, and FAK, through their surface functional groups. However, they are not selective for tumor cells and interact similarly with normal cells, although the strength of this interaction is weaker, or have an opposite effect on normal cells compared to the tumor cells. To the best of our knowledge, we observed for the first time a significant decrease in FAK expression at both mRNA and protein levels in U87 glioma cells treated with rGO/ATS and rGO/TUD flakes. Moreover, although the final effect is similar in normal and cancerous cells (i.e., reduced motility), it must follow different pathways because the expression of key proteins related to mobility (i.e., integrins, β-catenin, cadherin, and FAK) is not consistent in these cells. Further research should be carried out on a more advanced research model, which would verify whether changes in the expression of the studied molecules responsible for migration and invasiveness increase/decrease the virulence of these tumors.

## Figures and Tables

**Table 1:** Primers used to assess the expression of genes involved in cell adhesion and migration.

Genes	Forward Primers (5′ to 3′)	Reverse Primers (5′ to 3′)	References
*ITGA5*	TGCAGTGTGAGGCTGTGTACA	GTGGCCACCTGACGCTCT	[31]
*ITGB1*	GAAGGGTTGCCCTCCAGA	GCTTGAGCTTCTCTGCTGTT	[31]
*CDH2*	ACAGATGTGGACAGGATTGTGGGT	TATCCCGGCGTTTCATCCATACCA	[32]
*CTNNB1*	CCTATGCAGGGGTGGTCAAC	CGACCTGGAAAACGCCATCA	[33]
*PTK2*	CCCACCAGAGGAGTATGTCC	CCCAGGTCAGAGTTCAATAG	[34]
*rpl13a*	CATAGGAAGCTGGGAGCAAG	GCCCTCCAATCAGTCTTCTG	[30]

**Table 2:** Analysis of U87 and Hs5 cell motility in co-culture treated with graphene (GN) and reduced graphene oxide (rGO) expressed in μm/s. Statistical significance between the control and the treated cells is indicated by an asterisk and was assessed using Bonferroni’s multiple comparisons test. Differences with *p* < 0.05 were considered statistically significant. One asterisk (*) indicates *p* < 0.05, three asterisk (***) indicates *p* < 0.001 and four asterisks (****) indicate *p* < 0.0001.

	Motility (μm/s)			
	C	GN/ExF	rGO/ATS	rGO/TUD
U87	11.73	10.92	5.72 ****	8.11 ***
Hs5	10.08	8.73 *	6.44 ****	7.22 ***

**Table 3:** Analysis of U87 and Hs5 cell motility inhibition in co-culture treated with graphene (GN) and reduced graphene oxide (rGO) expressed in percentage of mobility inhibition (%). Statistical significance between the control and the treated cells is indicated by an asterisk and was assessed using Bonferroni’s multiple comparisons test. Differences with *p* < 0.05 were considered statistically significant. One asterisk (*) indicates *p* < 0.05, three asterisk (***) indicates *p* < 0.001 and four asterisks (****) indicate *p* < 0.0001.

	Percentage of Mobility Inhibition (%)			
	C	GN/ExF	rGO/ATS	rGO/TUD
U87	0	6.86	51.20 ****	30.86 ***
Hs5	0	13.41 *	36.06 ****	28.35 ***

**Figure A1:**
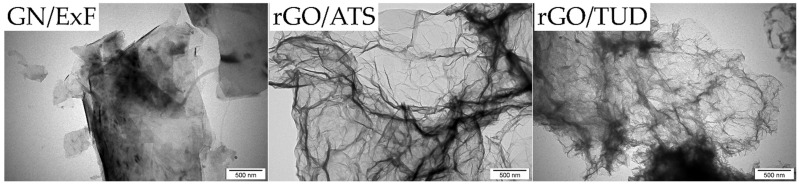
Transmission electron microscopy (TEM) images. Images of GN/ExF flakes and two types of rGO (rGO/ATS, and rGO/TUD). Abbreviations: rGO, reduced graphene oxide; GN, graphene. Notes: All necessary analyses regarding the characteristics of graphene and reduced graphene oxides (including TEM, SEM, FTIR, Raman, etc.) can be found in the works of Szczepaniak et al. 2018, 2021 [29,49].

## Data Availability

The data presented in this study are available on request to the corresponding author.
